# B-SAFE: Blockchain-Enabled Security Architecture for Connected Vehicle Fog Environment †

**DOI:** 10.3390/s24051515

**Published:** 2024-02-26

**Authors:** Priyanka Gaba, Ram Shringar Raw, Omprakash Kaiwartya, Mohammad Aljaidi

**Affiliations:** 1 School of Computer Science Engineering & Technology, Bennett University, Greater Noida 201310, India; priyanka@bennett.edu.in; 2 School of Computing Science & Engineering, Netaji Subhas University of Technology, New Delhi 110031, India; rsrao@aiactr.ac.in; 3 Department of Computer Science, Nottingham Trent University, Nottingham NG11 8NS, UK; 4 Computing and Informatics Research Centre, Nottingham Trent University, Nottingham NG11 8NS, UK; 5 Computer Science Department, Faculty of Information Technology, Zarqa University, Zarqa 13110, Jordan; mjaidi@zu.edu.jo

**Keywords:** blockchain, vehicular networks, security, online vehicle hijacking

## Abstract

Vehicles are no longer stand-alone mechanical entities due to the advancements in vehicle-to-vehicle (V2V) and vehicle-to-infrastructure (V2I) communication-centric Internet of Connected Vehicles (IoV) frameworks. However, the advancement in connected vehicles leads to another serious security threat, online vehicle hijacking, where the steering control of vehicles can be hacked online. The feasibility of traditional security solutions in IoV environments is very limited, considering the intermittent network connectivity to cloud servers and vehicle-centric computing capability constraints. In this context, this paper presents a Blockchain-enabled Security Architecture for a connected vehicular Fog networking Environment (B-SAFE). Firstly, blockchain security and vehicular fog networking are introduced as preliminaries of the framework. Secondly, a three-layer architecture of B-SAFE is presented, focusing on vehicular communication, blockchain at fog nodes, and the cloud as trust and reward management for vehicles. Thirdly, details of the blockchain implementation at fog nodes is presented, along with a flowchart and algorithm. The performance of the evaluation of the proposed framework B-SAFE attests to the benefits in terms of trust, reward points, and threshold calculation.

## 1. Introduction

The Internet of Connected Vehicles (IoV) framework is growing significantly as a roadside next-generation wireless system [1]. It is due to the recent advancements in sensor and communication technologies, enabling a range of vehicular interactions, including vehicle-to-vehicle, vehicle-to-roadside units, vehicle-to-mobile-infrastructure, vehicle-to-sensors, and vehicle-to-personal devices. According to a recent report by the International Data Corporation (IDC), the number of connected vehicles worldwide is projected to increase up to 76.3 million units by 2023, with an approximately 16.8% annual growth rate [2]. The global IoV automobile market, in terms of driver- and passenger-centric solutions, is predicted to grow to USD 166 billion by 2025. However, the major market for IoV is consumer-centric cars and green-transport-oriented public transport [3]. On the other hand, the IoV market has the potential to expand far beyond the car consumer market to industrial heavy vehicles and delivery vehicles by revolutionizing infrastructure manufacturing in cities and urban on-demand next day deliveries [4].

**Figure 1:**
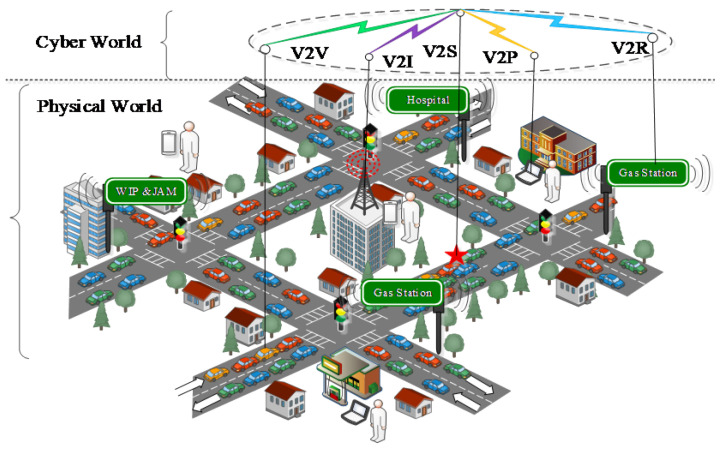
The cyber and physical world in IoV environments considering Vehicle-to-Vehicle (V2V), Vehicle-to-Infrastructure (V2I), Vehicle-to-Personal devices (V2P), Vehicle-to-Roadside (V2R), and Vehicle-to-Sensors (V2S) communication.

Online vehicle hijacking is an increasing concern, with the continuous development of IoV for safety and efficiency-oriented sustainability in transportation (see Figure 1) [5]. A few examples of online vehicle hijacking include unauthorized access to steering wheels, disabling brakes and other control wires, unlocking doors, engine disruption, road map forging, identity and location manipulation, denial of traffic services, and vehicle tracking [6]. The vehicular security threat is quite similar to the security threat in computer networks, which have been witnessed many times in the form of unauthorized system access, application hijacking, and unauthenticated data manipulation on a large scale, targeting individuals, organizations, or even entire systems of a country [7]. However, the consequences of vehicular security threats are quite different and more severe than those of system security threats in road-traffic-oriented conditions. A single-vehicle hijacking might lead to a deadly accident, causing the deaths of many people on the road [8].

Traditional cryptography-oriented security solutions have very limited feasibility in IoV environments, considering the constraints in vehicular traffic environments [9]. Few implementations of traditional security architecture for vehicular traffic environments have been explored in the literature. A cross-layer vehicular message authentication technique has been suggested, considering a location-centric cryptographic identity as a digital signature in the IoV environment [10]. Similarly, a group of messages as an authentication method has been suggested for reducing the delay in verifying a larger traffic flow of messages rather than a single message [11]. Improvements in these types of cryptography-based vehicular message authentication techniques have been investigated, considering single as well as batch or groups of messages [12]. The improvement has focused on providing a security guarantee against chosen-identity and no-message attacks as well as chosen-identity and chosen-message attacks. These authentications were based on bilinear pairing, which is a challenging cryptographic operation in vehicular traffic environments. To enhance the bilinear-pairing-based cryptographic vehicular message authentication, a conditional authentication technique that does not rely on the bilinear pairing of vehicular nodes for distributed authentication has been suggested [13].

Non-cryptographic security solutions have been explored, considering the challenges of the centralized execution of cryptographic operations in vehicular traffic environments. These non-cryptography-oriented security architectures have been majorly based on cooperative trust or belief management in vehicular networking environments. A non-cryptographic location verification technique has been suggested based on a transferable cooperative belief model for vehicular traffic environments [14]. Similarly, a trustworthy event information dissemination framework has been suggested, considering the truth-telling probability of neighboring vehicles and, in particular, without using cryptography [15]. Another trusted and reputable management framework has been suggested for detecting vehicles transmitting malicious or bogus messages in intelligent traffic information dissemination using unmanned aerial vehicles or drones [16]. However, these trust or belief-centric non-cryptographic security solutions have limited applicability in traffic applications that require high reliability. On the contrary, recent advancements in blockchain-enabled security solutions [17] have potential in vehicular fog networking scenarios to effectively address security concerns in IoV environments [18].

In this context, this paper presents a Blockchain-enabled Security Architecture for a connected vehicular Fog networking Environment (B-SAFE). Since blockchain is a decentralized and distributed transaction-oriented security architecture, the framework provides a potential and viable security solution for IoV environments. The contributions of this paper are as follows:

- Firstly, blockchain security and vehicular fog networking are introduced as preliminaries of the security framework.
- Secondly, a three-layered architecture of B-SAFE is presented, focusing on vehicular communication, blockchain operations at fog nodes, and the cloud as a trust and reward management for vehicles.
- Thirdly, details of the phase-wise blockchain implementation at the fog nodes are presented, along with a flowchart and algorithm.
- Finally, the performance of the evaluation of the proposed framework B-SAFE attests to the benefits of its use in terms of trust factor, reward points, and threshold calculation.

The rest of this paper is organized as follows. In Section 2, the related literature on security frameworks for IoV is critically reviewed. Section 3 introduces the blockchain security and vehicular framework for networking as preliminaries. Section 4 presents the details of the proposed B-SAFE framework. Section 5 discusses the performance evaluation of B-SAFE, followed by conclusions presented in Section 6.

## 2. Related Work

Vehicular Fog Networking (VFN) is the integration of fog computing and Vehicular Ad Hoc Networking (VANET) for providing cloud services to nearby vehicles to deal with timeliness and latency issues [19]. Vehicular fog computing suffers from security and privacy issues [20]. Another problem is that, although the fog and cloud service providers are trusted entities, vehicles in VFN are often not comfortable with sharing personal information with unknown fog devices [21]. In VFN, vehicles are connected to the internet, which is also a big reason for cyber-attacks. Blockchain, a distributed, decentralized, immutable, consensus-based network, could be considered an effective solution to overcome the timeliness, latency, and cyber-attack problems of VFN [22]. Work performed by various authors related to the blockchain with VANET or VFN is discussed here.

The purpose of VANET is to provide the facility to share messages among vehicles. The challenge here is that there is a need for a trusted entity to store and forward such messages. Another challenge is the vehicle might not be willing to participate in the generation and distribution of announcement messages unless it receives a benefit from such participation. To deal with this problem and provide secure communication, authors have suggested a blockchain-enabled secure data-sharing system for the Internet of Vehicles (IoVs) using a parent and auxiliary blockchain to store the messages by different entities from different regions [23]. To maintain privacy, a fair blind signature, threshold secret sharing, and punish–reward mechanisms are applied to encourage participation. In the IoVs, because of their highly dynamic nature, vehicles generally move at a high speed on the road. Traditional models that work for cross-data-center authentication are not appropriate enough and provide a delayed output; to overcome this drawback, researchers have suggested a Blockchain-Assisted Lightweight Authentication (BALA) for vehicular fog services through non-interactivity between the vehicles, service manager, and authentication process [24]. It also provides vehicles with the flexibility to authenticate but not while entering a new data center, which can also protect their privacy and make the system lightweight.

To improve security issues faced by the IoVs, a new type of blockchain framework has been explored to enable the secure transmission of data [25]. For this, the researchers created a local public blockchain that stored the trustworthiness of a node and a message in a ledger. The authors in [26] have pointed out the issue that there is a lack of inspiration in vehicles to forward alert messages and issues with forwarding them without revealing their identities. They suggested an efficient blockchain-based privacy-preserving incentive announcement network that allows vehicles to work anonymously in the network and also facilitates them with incentives for their work. A two-phase trust management system has been investigated in the IoVs [27]. First, for secure message transmissions, the privacy preservation model is applied with a key, which is used for the sanitization process; this algorithm is the “Sea Lion Explored-Whale Optimization Algorithm”. Second, for evaluating node trustworthiness, rule- and machine-learning-based processes are applied. To build trust in reliable vehicles and maintain their privacy, the authors in [28] suggested the system “BARS”, which is a blockchain-based model for the IoVs. It utilized proof of presence and absence. To hide real-identity communication, they used public keys as pseudonyms. They presented a reputation evaluation algorithm to avoid fake messages, which is based on past communications and judgments regarding the vehicles.

In order to facilitate the secure interchange and storage of data within in-vehicle edge networks, the researchers in [29] employed a combination of consortium blockchain and smart contract technologies. These technologies serve to inhibit the unlawful sharing of information. Additionally, a reputation-based data-sharing approach was devised by the researchers to ensure that the vehicles consistently contributed high-quality data.

The authors in [30] suggested a multi-access edge computing paradigm for VANET and established a model by applying the kNN algorithm. This scheme reduces the communication message latency and enhances the routing path. Using edge computing enabled through blockchain, the authors in [31] created software-defined fault tolerance and quality-of-service-aware IoT-based vehicular networks, reducing the total communication time and message failure fault tolerance and increasing safe service delivery for VANET.

## 3. Preliminary and Proposed Solution

The blockchain security network was originally initiated with Bitcoin, a digital currency that uses a decentralized network. It has evolved as a strong platform for the decentralization of security architecture in every field [32]. Bitcoin, Litecoin, and Dash are Blockchain 1.0 technologies. Blockchain 2.0 technology evolved to deal with the ownership issues related to properties and contracts. Examples of Blockchain 2.0 are Ethereum, NEO, and QTUM. Blockchain 3.0 evolved to use decentralized storage and communications, such as with DApps. Blockchain 4.0 evolved to make it usable for diverse industries. This advanced blockchain technology has shown its impact not only in finance applications but also in non-financial applications that cover many dimensions of life [33].

Blockchain technology is one of three types of implementation frameworks [34]. The first is a public blockchain, where anyone can be involved in the process of verifying and obtaining a consensus. Examples of public blockchains are Bitcoin and Ethereum. The second type is consortium blockchains, which can be open or confidential, and so they seem to be moderately decentralized. Examples of consortium blockchains are R3CEV and Hyperledger. The third type is a private blockchain, which is completely confidential, permitting only the members of a company. Based on who can publish blocks, a blockchain network is considered “permission less” if anybody in the network can publish and “permissioned” if only specifically defined entities can publish blocks [35]. Blockchain technology uses features like peer-to-peer networks, cryptography, smart contracts, consensus algorithms, etc. The smart contract is a prewritten program stored as a bytecode that executes automatically to apply the logic of the application. Any node in the network can participate in the process of block creation and validation and earn incentives out of it.

### 3.1. Block Data Elements—Header and Body

**Figure 2:**
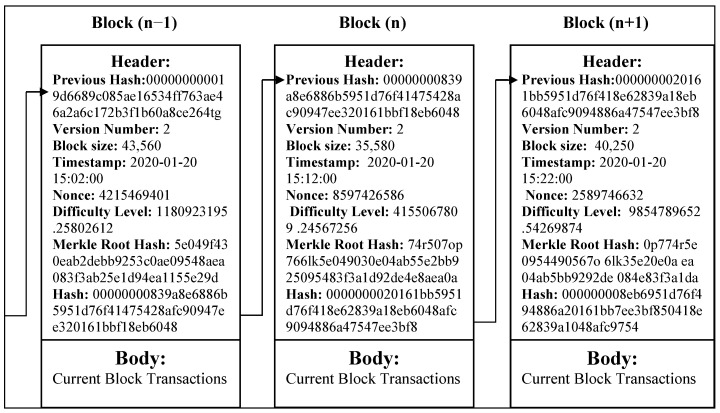
Data elements of a block in blockchain.

A blockchain is a list of connected blocks where each block is a collection of multiple transactions that may be from multiple users of the network [36]. The first block in the blockchain network is known as the Genesis block. An example of data in the blocks is shown in Figure 2. Each block consists of two parts: a header section and a body section, which we have explained below. The header contains basic information related to the block, such as a Previous Hash, which is kept to maintain the connection and chronology between each block. The version number is related to the software, although this is not very relevant in most cases. However, it may be used by the miner to signal which protocol decision it supports. The size of the block is represented in bytes, which are stored in the block size field.

The timestamp field is used to record the time in seconds in which the block was created. Nonce, a short form for “number only used once”, is a number added to a hashed block to meet the constraints of the difficulty level when rehashed. The difficulty level field indicates the length of the goal of the minimum bits needed to claim validity. This is inversely proportional to the easiness of finding the hash. The Merkle Root Hash is related to mathematics scientist Ralph Merkle. The target of this tree is to represent huge amounts of information using a single hash. Each transaction is like a leaf of the tree, which is hashed and merged with another transaction’s hash to obtain a single hash [37]. In a similar way, the set of the two transactions hashed are merged, which are further hashed to obtain a final single hash that acts like the root of the tree. The hash field contains the current block’s hash, which is calculated based on the above field values. The body contains a list of transactions involved in the block, events, or any other data.

### 3.2. Features of Blockchain

The trust required within a blockchain network in any of the applications is facilitated by the following features of the blockchain network, which is even, in fact, without the presence of trusted mediators [38]. Decentralized means a single authority is not responsible for the maintenance of the complete network, but rather, multiple nodes that are all involved are responsible. A distributed ledger means a public ledger is open to all, providing all the necessary information regarding transactions and users of the network except for private and federated blockchain. Immutable means no one can change or modify it. In blockchain, immutable means that once the block is added to the ledger, no one can change anything because the hash of the node will change, and so all the subsequent nodes will change. Secure means the security of the blockchain network is maintained by using an encryption technique. To double-fold security, cryptography is also used to hash all the information. Consensus means to run a system smoothly; a consensus algorithm is essential where a huge number of nodes may be vehicular nodes, such as in the case of VANET, where they are validating a block. It is also necessary for the decentralization feature of every blockchain. Different types of consensus algorithms, like Proof-of-Work, Proof-of-Stake, etc., may be used.

### 3.3. Operations in Blockchain

**Figure 3:**
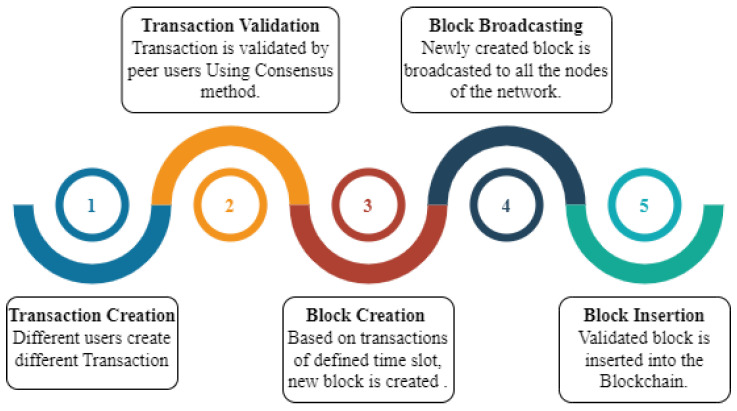
Major working steps in blockchain implementation.

Blockchain consists of multiple blocks linked to each other using a hash. Each block consists of multiple transactions that happen within a particular defined duration. The transaction is the data exchange between two or multiple nodes [39]. A working blockchain system follows the sequence shown in Figure 3 and is explained below.

It starts with step one, which is the initiation of a transaction request from a node of the network that signs the transaction with its private key to generate a unique digital signature so that nobody can modify it. In step two, the transaction of the network is validated by peer nodes or users by any of the consensus methods, and a validated transaction obtains a place in the ledger. Every transaction has a timestamp and unique ID. In step three, all the verified transactions that happened over a defined period are combined to create a block that has a unique fixed-length hash that is constructed using its various attributes. In step four, the newly created block is broadcast to all the nodes. In step five, the validated block is added at the end of the existing blockchain, and the ledger is also modified.

### 3.4. Vehicular Fog Networking

The IoVs are one of the substantial applications of fog computing, and this integration is known as a Vehicular Fog Network (VFN) [40]. A VFN gives the advantage of low latency, a lower network bandwidth requirement, and security, and is more reliable since vehicles do not need to communicate data to the cloud. In the case of a VFN, any static node like a router, switch, base station, RSU, or dynamic node like a vehicle could act as a fog device. A fog device has an unutilized infrastructure, so it can be rented out to the required vehicles for storage and computing. Apart from this, the fog is also involved in the process of segregating data, forwarding it, or making real-time decisions for vehicular communication [41]. Despite sending complete data to the cloud, the fog sends data required for future analysis.

In a VFN, a set of smart vehicles in close proximity to one another might establish a vehicular fog network by connecting to one another via a specialized short-range communication system operating in the 5.9 GHz band with a 75 MHz spectrum range. If vehicles are the source of this interaction, then both the vehicle owners and service providers will gain an advantage [40]. Depending on the needs of the system, communication and computation could take place between moving and stationary vehicles. Fog computing also supports the various services provided by VANET, such as routing, offloading, security, privacy, and message dissemination [42].

**Figure 4:**
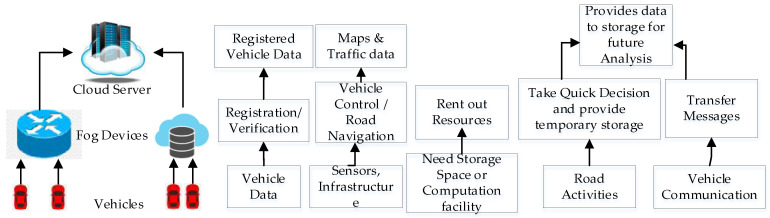
Components of VFN with its functionalities.

A VFN deals with the mobility management of vehicles between different fog servers to maintain the quality of service and provide essential solutions to the network [43]. The components of a VFN, with its functionalities and their connections, are shown in Figure 4 [44]. Various authors have proposed their architecture, algorithms, and ideas for a VFN to make the system efficient. VFNs still face the challenge of security, which could be handled by applying blockchain to it. Our proposed system is based on blockchain to make the VFN system more secure. Notations used in this paper are mentioned in Table 1.

### 3.5. Proposed Solution

The shortcomings of traditional VANET systems and cloud computing have spurred the transition towards fog computing and blockchain technology to tackle existing challenges. In response, we introduce a secure and efficient data-sharing solution that adheres to privacy protocols, effectively addressing the issues plaguing VANET. Our proposed approach meets key requirements for a reliable and privacy-compliant solution.

- Firstly, in order to guard against data manipulation and preserve data integrity, this system integrates a blockchain-based data storage approach. The transmission of vehicle data leads to the creation of a blockchain transaction, which is subsequently included in a block. The utilization of the transaction address facilitates the authentication of the vehicle’s identity, hence obviating the necessity for a signature and enhancing the dependability of the network. Furthermore, this serves to protect Vehicular Ad Hoc Networks (VANETs) from potential privacy breaches and authentication threats.
- Secondly, an additional challenge within VANET lies in the reliance on vehicles to blindly accept reported events without verifying their accuracy. To enhance the precision of shared data pertaining to incidents, the system incorporates nearby vehicles in the vicinity of the event to assess the validity of the provided event data. By involving proximate vehicles in making judgments regarding event correctness, this approach aims to bolster the reliability of information shared among vehicles in the network.
- Thirdly, VANET is integrated with fog computing to extend cloud-like features to the network edge for enhanced speed and efficiency, whereas traditional cloud systems suffer from drawbacks such as latency and dependency on centralized servers.
- Fourthly, a vehicle may lack the desire to take an active role in the confirmation of an incident that occurred earlier on the road. Thus, to motivate vehicles to be involved in giving information regarding event occurrence or giving judgment for validating that event data, incentives are provided to these vehicles in the form of reward points.
- Lastly, the VANET system does not even maintain the details of each vehicle, nor does it assess its reliability. In the proposed architecture, the trustworthiness of each vehicle is also evaluated and stored in the system, which analyzes the required number of vehicles for judgment. This factor is crucial for computational complexity and response time.

## 4. Blockchain-Enabled Security for Vehicular Fog Network

In this section, the blockchain security framework is presented with vehicular fog computing as a new vehicular network architecture B-SAFE. The vehicular network architecture combined with fog computing provides features of cloud computing at the edge of the network and, therefore, makes blockchain security transactions faster. This system is referred to as Vehicular Fog Network (VFN). To make VFN more secure by storing the value of reward points and trustworthiness of vehicles in a traffic environment, the blockchain concept is applied to VFN. Moreover, the blockchain concept, together with fog computing, could resolve the major security concerns in an IoV environment. Therefore, we have integrated blockchain concepts with VFN and proposed a new framework called a Blockchain-enabled Security Architecture for a vehicular Fog network Environment (B-SAFE).

### 4.1. Overview of B-SAFE

**Figure 5:**
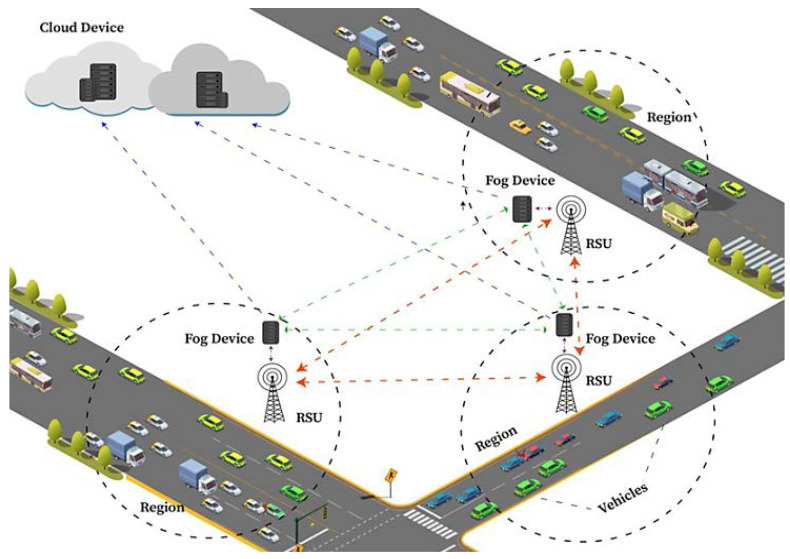
Working overview of B-SAFE.

The B-SAFE consists of static and mobile vehicles on the road, RSUs, fog devices, and the cloud, as shown in Figure 5. The network diagram defines the local in-vehicle domain through fog devices and RSUs to access the whole intelligence of the network managed by the B-SAFE administrator. A specific diameter area is under a region that has a defined RSU belonging to that region. All the vehicles in that region can interact with each other and with the RSU of that region as well. For inter-region communications, RSUs can interact with each other. Every region also has a fog device attached to the RSU to provide cloud services to that region. All fog devices can interact with each other and the cloud to store permanent data that could be used later. Fog devices are also responsible for blockchain creation and propagation.

### 4.2. Network Architecture of B-SAFE

**Figure 6:**
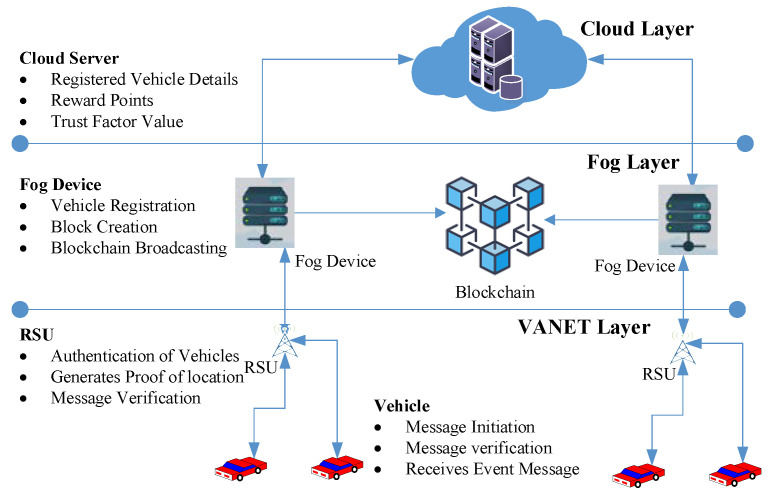
Layered Architecture of B-SAFE.

The proposed B-SAFE will create a peer-to-peer, protected, and decentralized network for inter-vehicle communication and store the data on an immutable blockchain [45]. The purpose of this network is to provide two functionalities. The first is to design a message verification process for RSUs using neighboring vehicles. The second is to design a reward- and punishment-based mechanism that provides incentives to trustworthy vehicles for motivation and punishments to faulty vehicles for discouraging fake messages. These are stored as reward points for every vehicle. These reward points could be redeemed later to provide a benefit to the vehicle. One more factor is attached to the vehicles to judge the trustworthiness of the vehicle, which is based on the correctness of their sent and verified messages, and this factor is termed the Trust factor (T_f_) value. This value helps the system to judge the behavior of the vehicle. To make this functioning possible, each component in the network has its defined role and responsibilities. The layered architecture in Figure 6 shows all the entities and their functionalities. A detailed explanation of each layer is presented in the following subsections.

#### 4.2.1. VANET Layer

This layer basically describes on-road vehicular communication in traffic environments along with roadside and mobile network infrastructure. Vehicles moving on the road have the capability of detecting events related to traffic, can process and store information using an On-Board Unit (OBU) installed on the vehicle, and can also communicate with nearby vehicles using Long Term Evolution (LTE) or Dedicated Short-Range Communications (DSRC). The main entity in the VANET system is the vehicle that plays the following roles. As an initiation of event messages, a vehicle that either undergoes an event on-road or witnesses any event could report that event to other vehicles or the RSU. As verification of event messages, messages sent from every vehicle cannot be assumed truthful since malicious vehicles could generate a fake message for their benefit, to disturb others, to clear the path, or maybe for some other reason. Hence, messages being initiated should be verified first before being added to the blockchain. Vehicles that are near the event location could give information regarding the message’s correctness. As the receiver of an event message, the event message is meant for other vehicles that are approaching that road so that they can take corresponding actions. The message is transferred to such vehicles that could be affected by this event.

The RSUs are considered computing devices that can act as access points located on the roadside to provide connectivity to moving vehicles and, therefore, enable a smooth traffic flow by giving responses to emergency events. They belong to a single region and perform the management tasks of the vehicles of those regions. Other than these tasks, RSUs also accomplish the following tasks in B-SAFE. In the authentication of vehicles, RSUs authenticate the vehicles belonging to its region that are participating in event message communication by checking their identity details and digital signature attached to a message. In Proof of Location (PoL), RSUs capture the location of the vehicle that is initiating any event message and verify it with the location of the event. If the matches provide a PoL certificate to initiate a vehicle, then the RSUs discard the event message since it could be fake. In message verification, RSUs also perform the task of message verification from other nearby vehicles to analyze its correctness. RSUs need to find nearby vehicles by calculating inter-vehicle distance with or without using GPS [46]. In fog node communication, they forward the validated messages to the fog to create a blockchain. In the transmission of messages, verified event messages should be forwarded to another region’s RSU so that the message can be forwarded as an alert message to vehicles in its regions.

#### 4.2.2. Fog Layer

Fog devices are considered and deployed alongside the road everywhere near the vehicles to provide cloud-like features to make the system work faster and more efficiently. Examples of roadside fog devices include RSUs, in-vehicle phones, laptops or any computing devices, routers, embedded servers, and video surveillance cameras, and they could also be dedicated vehicles. The responsibilities of fog devices in blockchain-enabled IoVs are listed here. The registration of vehicles, which is the one-time registration of the newly added vehicle to the network, provides a unique public key and private key pair for communication along with a unique digital signature to store all these details with the vehicle ID as well as to the cloud. Transfer event messages, which are to transfer messages received from the RSU to other fogs of nearby regions so that immediate actions could be taken by the fog nodes of nearby regions to somehow control the traffic. Block creation combines a defined maximum allowed number of transactions over time, creates a block, and also helps in mining a block. Blockchain updating is when the newly created block is added to the blockchain, and the update is broadcast to all the nodes of the network. Communication with the cloud is where fog nodes transfer all the details related to the vehicles to the cloud for future access and also update the trust level scorecard of every vehicle from time to time and when any change occurs.

#### 4.2.3. Cloud Layer

The cloud layer is the topmost layer in the layered architecture of B-SAFE. This layer provides cloud services via the fog layer. The cloud acts as a reservoir of the data related to blockchain-enabled IoVs, which need not be stored anywhere else on multiple servers; just one location will be enough and, therefore, can be accessed anytime from anywhere. It mainly stores the details of registered vehicles, like key pairs, their identity, and their digital signature. These details are stored when a new vehicle is registered and will then start communication in the network. These details are accessed from time to time whenever there is a need to verify a vehicle and to prove that it is a registered vehicle of the network. One more possible usage is to extract details when there is a need to track a malicious vehicle.

### 4.3. Phases of B-SAFE

**Figure 7:**
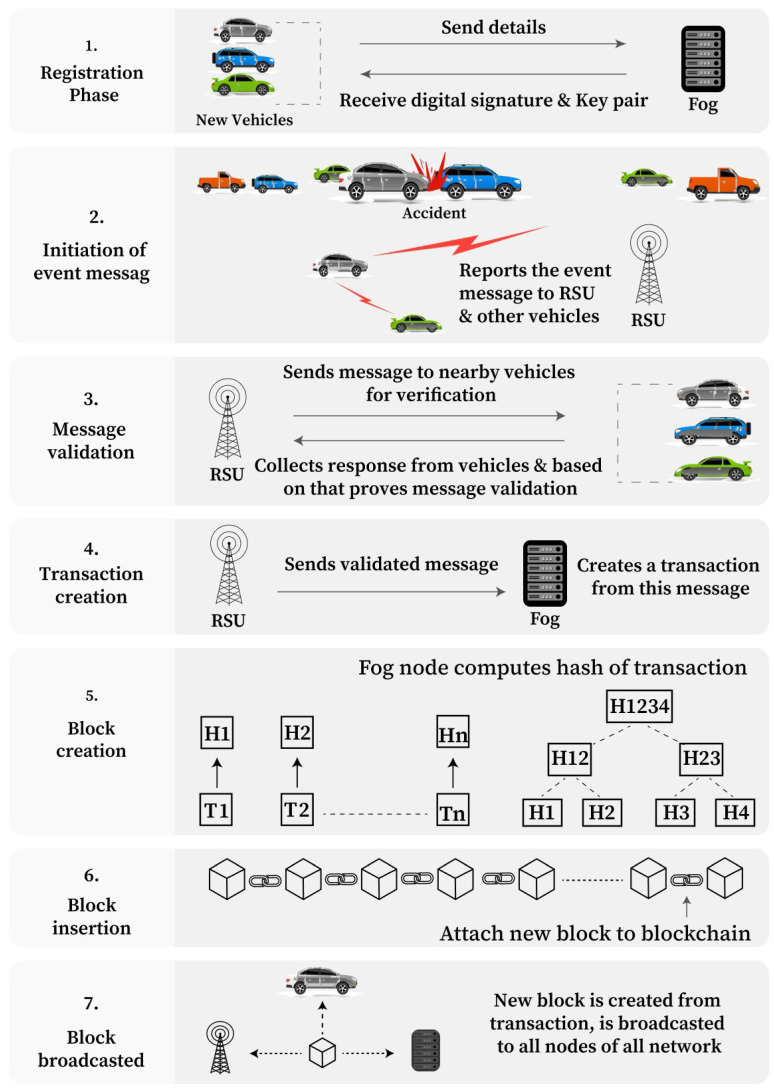
Major operation phases of blockchain implementation in B-SAFE.

This section presents the various blockchain operation phases involved in B-SAFE. These operation phases are shown in Figure 7.

#### 4.3.1. Registration Phase

The very first phase is executed just once during the whole lifecycle of B-SAFE for every vehicle. This phase chooses a trusted fog node that will be used for all further communications of that region and work for blockchain development. These fog nodes will now be responsible for registering the vehicles of the network for which the details of the vehicle are stored and will be issued a private–public key pair, which will be used by the vehicle for secure communication in the IoVs and a unique digital signature to prove the authentication of the sender node. The reward point and trust factor values are initialized to zero.

#### 4.3.2. Initiation of the Event Message

Real-time events like accidents, fires, traffic light failures, vehicle breakdowns, roads under construction, or areas of huge traffic are monitored and could be reported by vehicles that saw the event or are nearby vehicles and also by the RSU.

#### 4.3.3. Message Validation

Each message is considered a transaction for the block, but before adding it to the block, it must be validated because every node that is initiating an event message, even though it is registered on the network, cannot be trusted. RSU is responsible for checking the correctness of an event message in our proposed network. RSU first checks whether the location of the event matches the current location of the Initiating Vehicle (IV). If both do not match, then the message is definitely fake, so it is discarded, and the IV is punished for the fake message. If both match, then the RSU provides a location certificate to the vehicle and proceeds to verify the message. For this purpose, the RSU identifies vehicles near that event location and sends a message to them to verify the same event.

**Figure 8:**
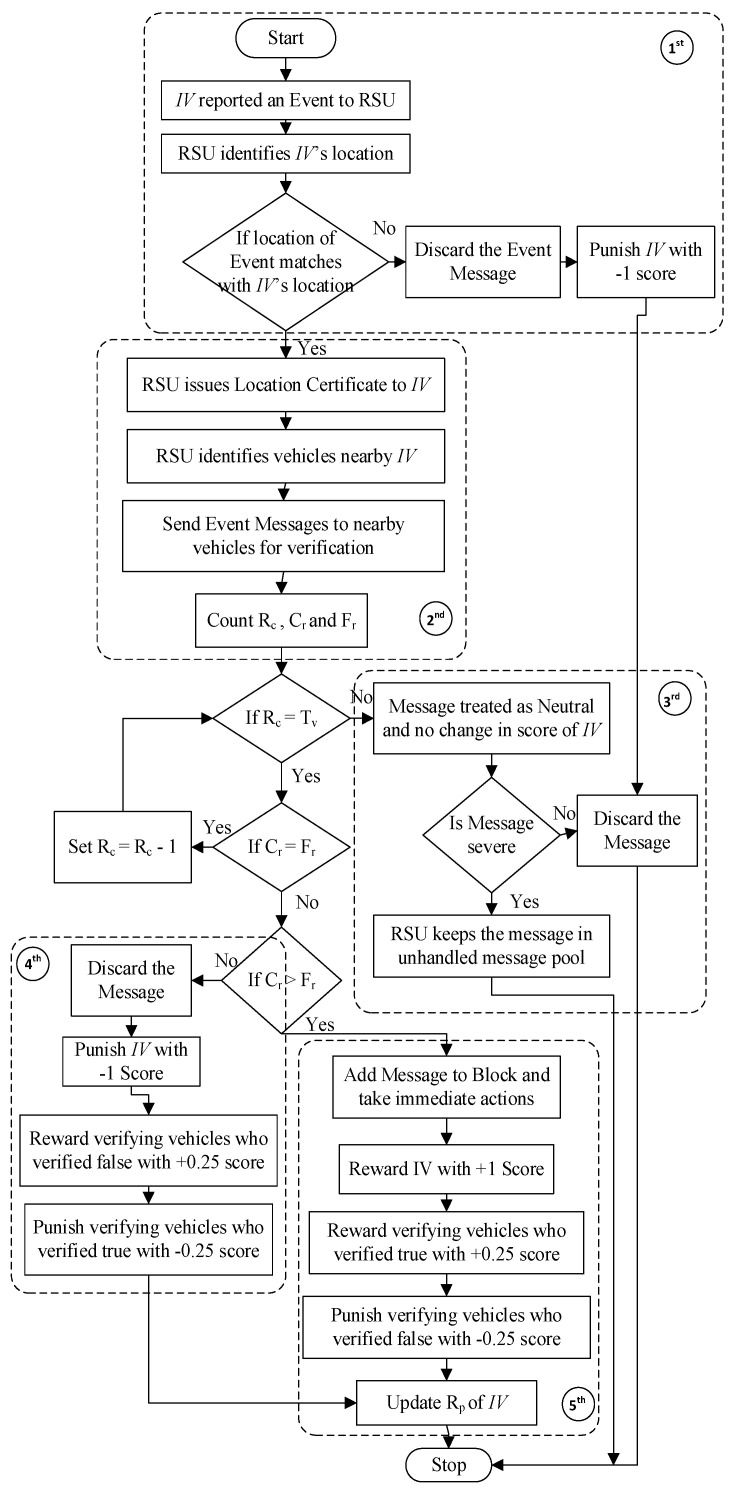
Flowchart of Message validation in B-SAFE.

The flowchart depicting this phase is shown in Figure 8. A Threshold Value (T_v_) is calculated (see Section 5) based on the number of nearby vehicles and the Average Trust factor (AT_f_) value of those vehicles. The RSU waits until it receives responses from the number of vehicles equal to T_v_. Then, a decision is taken based on the number of received replies saying “Correct Response” (C_r_) or “Fake Response” (F_r_), which is like a counter and is compared. If both C_r_ and F_r_ values are equal, then one more reply, either in favor or against, is needed to make a decision. If the value of F_r_ is more than C_r_, then the message is declared fake. Therefore, the message would be discarded and the IV punished. Punishment is also given to those vehicles that provide a response saying the message is correct. In turn, the vehicles that gave a response saying the message was fake are rewarded. If the value of C_r_ is more than F_r_, then the message is proven valid. In this case, the reward is given to the IV for contributing a correct message. The vehicles that responded with the message as correct are also rewarded. In turn, vehicles that send a message saying the event is fake are liable to punishment. The score of every involved vehicle is updated at the end.

#### 4.3.4. Transaction Creation

Messages sent from different vehicles are validated by the RSU, as expressed in the above phase, which will result in either the message being valid or invalid. The valid message is forwarded to a fog device by the RSU to create a transaction of the block. Every transaction has a message of the event attached to it, a unique ID, and a timestamp, which signifies the time of occurrence of the event. The most important task after transaction validation is to forward the event message to all the intended sources so that immediate corresponding action can be taken as per the requirement. The transaction is to be forwarded to connected-region fog devices that will send an alert message to the RSU and then to vehicles approaching the event location.

#### 4.3.5. Block Creation

**Figure 9:**
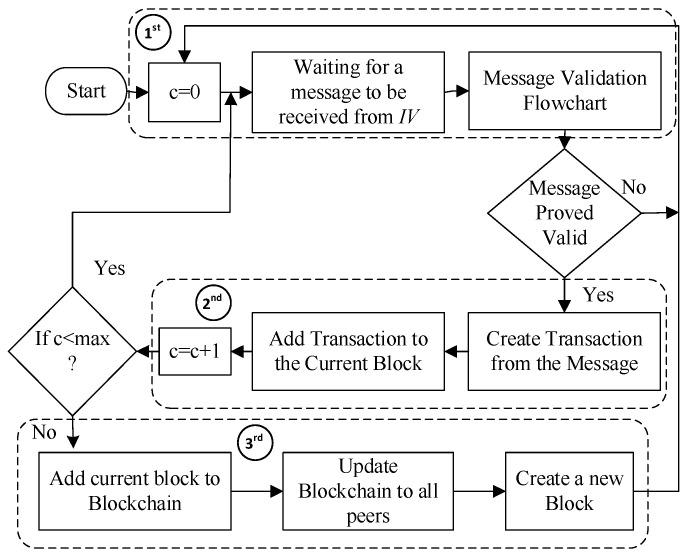
Flowchart of block creation in B-SAFE.

A block consists of a defined number of valid transactions. The maximum allowed number of transactions is denoted by a “max”. Validated transactions are added to the block until the total number of transactions in a block does not reach max value, and when it reaches it, a new block is created. The same is depicted in the flowchart shown in Figure 9. To store all transactions in a single block, the Merkle Root Hash tree is created, which can store multiple transactions in the form of a tree, where the leaf node contains an individual transaction hash. Two transaction hashes are combined to make a single merged hash, and this process continues for all transactions. Then, two merged hashes are combined to form a hash again, and the whole process is repeated until a single hash is created, which is the root of the tree and known as a Merkle Root Hash. This hash is combined with other attributes: the version number, block size, timestamp, nonce, difficulty level, and hash of the previous block. These attributes are merged and form a hash of the current block. The body part of the block contains a list of transactions, event data, and other records related to the transactions. One more blockchain is created here to store the behavioral characteristics of each vehicle in the form of a reward point and trust factor. These values can be accessed anytime from anywhere to judge a particular vehicle.

#### 4.3.6. Block Insertion

Two blockchains are created for the proposed network environment, where the first block of each blockchain is known as the Genesis block. All further created blocks are attached after that using the hash value of the previous block. The newly created block is attached at the end of the current blockchain.

#### 4.3.7. Block Broadcasting

The newly created block is broadcasted to all the nodes of the network so that everyone will have the updated copy of the blockchain, which is now used further, and the next block will be inserted after that only. Here, the nodes of the network mean that all the vehicles, the RSU, and the fog containing a copy of the blockchain will be updated about the new block.

#### 4.3.8. Algorithm and Its Description for B-SAFE

The B-SAFE creates a block in seven phases, as discussed above in Figure 7. Algorithm 1 for the message verification phases is shown below. It starts with an Initiating Vehicle (IV) that will send an Event Message (EM) of any event with its complete details to the RSU. The RSU, on receiving the message, checks the current location of the IV and the Location of the event (L_e_). If both are not the same, then it simply discards the message, treating it as fake and punishing the IV by deducting one point from its Trust factor (T_f_); otherwise, the RSU issues a Location Certificate (LC) to the vehicle and identifies the vehicles moving near to that event location and creates a List of Nearby Vehicles (NBV_LIST). The RSU then sends the reported event message to the nearby vehicle list and asks for confirmation. Vehicles will either reply saying the message is correct or fake, and accordingly, the pointer for either C_r_ or F_r_ will be updated, also appending the vehicle into either the Correct Response Vehicles List (CRV_LIST) or Fake Response Vehicles List (FRV_LIST). This will be performed until the time elapsed is less than the Defined Allotted Time (D_t_) or the Response Counter (R_c_) is less than the decided Threshold Value (T_v_).
**Algorithm 1**: Message Verification Process**Input:** L_v,_ L_e_

**Output:** LC(IV), R_p,_ T_f_

**Process:**

Received EM from IVExtract L_v_ and L_e_If L_v_! = L_e_ then:  Set R_p_ (IV)− = 1, Set flag = 0, Discard the message, Exit.Issue LC(IV)Create NBV_LISTSend EM to NBV_LIST for confirmationSet R_c_, C_r_, F_r_, T = 0Start TCreate two empty lists CRV_LIST FRV_LISTRepeat while (R_c_ < T_v_ or T < D_t_)
Received VM from VN, and set R_c_ ++If VM is True then:set C_r_ ++, and append VN to CRV_LISTelse:set F_r_ ++, and append VN to FRV_LIST
if T = D_t_ then:  Set flag = 0, and Discard the message and Exit.if Cr > Fr then:
set C_EP_ (IV) = 1, set C_m_ (IV) ++ and set flag = 1for all VN in CRV_LISTset C_EP_ (VN) = 0.25 and set C_m_ (VN) ++for all VN in FRV_LIST:set C_EP_ (VN) = −0.25 and set F_m_(VN) ++
if C_r_ > Fr then:
set C_EP_ (IV) = −1, set F_m_ (IV) ++, and set flag = 0for all VN in CRV_LIST:set C_EP_ (VN) = −0.25 and set F_m_ (VN) ++for all VN in FRV_LIST:set C_EP_ (VN) = 0.25 and set C_m_ (VN) ++
else:
set T = 0 and repeat steps 10–13
Update the value of R_p_ and T_f_ for IV and all VN.Return flag

The RSU needs to decide the threshold value T_v_ for the number of replies each time, which depends on the number of nearby vehicles and the Average Trust factor (AT_f_) value of nearby vehicles discussed in Section 5. If the RSU does not obtain the desired number of replies within the defined allotted time (D_t_), it declares the message neutral because it is a real-time scenario; therefore, the RSU cannot wait for an indefinite period, and, in this case, nobody will be rewarded or punished. If the number of replies reaches a threshold value, the RSU will stop waiting for any more responses and will check that the message is proven correct or fake by comparing the values of the C_r_ and F_r_ counters. If the value of C_r_ is more than F_r_, then the message is proven correct and sent as a block transaction, and the required actions are taken by the RSU. In this case, the IV is rewarded with +1 reward points, and all those vehicles that gave a correct reply will also be rewarded with +0.25 reward points, but the punishment of −0.25 reward points will be given to those vehicles that sent a message saying it is fake.

On the contrary, if the value of F_r_ is more than C_r_, then the message is declared fake. The message does not require any further action and is not added to the blockchain. In this case, the IV is punished with −1 reward points, and all those vehicles that gave a reply stating that the message is correct will also be punished with −0.25 reward points. Vehicles that send a message saying it is fake will be rewarded with +0.25 reward points. If the values of C_r_ and F_r_ are equal, then the RSU needs at least one more reply to prove it correct or fake, and in such a situation, the RSU waits for another reply and then takes the decision. Notations used for this algorithm are mentioned in Table 1.

We want to clarify that we have adapted the traditional blockchain framework implementation for vehicular network scenarios by considering traffic environment constraints. For example, we have used a different concept in the proposed framework to verify traffic message communication with the help of neighboring vehicles. We have used the concept of positive and negative reward points, which is different from traditional implementation, so as to motivate vehicles to communicate truthfully in the framework.

## 5. Performance Evaluation

B-SAFE aims to support the IoVs using blockchain to keep the network secure for communication and store the vehicle’s parameters, which can be used to judge the trustworthiness of a vehicle. There are two parameters used for each vehicle: Reward Points (R_p_) and a Trust factor (T_f_). Both the parameters are initially set to zero and updated as and when there is any change in value. Both the values are stored on the blockchain network so that anyone can access it anytime from anywhere. Reward points are the incentives earned by a vehicle that can be later redeemed. The trust factor value can be used to judge the vehicle’s past behavior regarding communication with peer vehicles. In this section, we have defined some trustworthiness parameters and derived them mathematically. The symbols used for these parameters in mathematical analysis have been mentioned in Table 1.

### 5.1. Reward Points

A vehicle seems a little disinterested in participating in event message initiation and verification if it is not receiving any benefit in return. Therefore, to motivate or encourage the vehicles to participate, B-SAFE can pay them reward points for their truthful participation. To demotivate or discourage malicious vehicles from sending a fake message for their benefit, punishment can be given to them in the form of negative reward points. A reward point is like digital money that a vehicle can earn by participating in communication-related events on the road in B-SAFE. This digital money is stored on the cloud server. The future of this concept is that it could be used by a vehicle to pay parking charges, tolls, gas stations, restaurants, etc. A reward point for every vehicle is initially set to zero for new vehicles and is updated automatically with the change in the value of C_EP_ according to Equation (1):

(1) $$R_{P}=R_{P}+C_{EP}$$

Current Event Points (C_EP_) are calculated as follows:

IM: +1 for correct message reporting

  −1 for fake message reporting

VM: +0.25 for verifying messages correctly

  −0.25 for verifying message falsely

**Figure 10:**
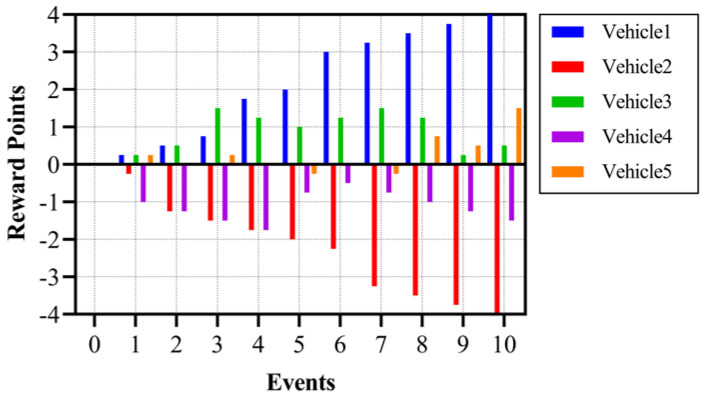
Reward points vs. event message.

To depict the change in reward points by initiating/verifying a correct or fake message, random data for five users are taken in Table 2, and there is a corresponding graph shown in Figure 10, which shows the change in reward points with every event. Events are taken from 1 to 10, and their corresponding value in the column indicates the vehicle number that initiated the event message. For five vehicles, the value of C_EP_ and total reward points, R_p_, after the event occurrence are given in the table. A value of 0 means that the vehicle never participated. A more positive value signifies greater participation in correct messages. On the contrary, negative reward points mean the vehicle participated in fake messages. Here, we have assumed that every vehicle is participating in every event, which, in real life, is not practical, but it is beneficial to show a variation in reward points.

The graph in Figure 10 depicts the variation in reward points of a vehicle with every event participation. An increasing value means truthful participation, and a decreasing value means fake participation. As vehicle one is always involved in truthful participation, the value of R_p_ increases with each event. Vehicle five, on the other hand, is always involved in fake participation, and therefore, the value of R_p_ decreases with each event.

### 5.2. Trust Factor

The Trust factor, T_f_, is the basis for judging the trustworthiness of a vehicle. Trustworthiness is based on the correctness of an initiated or verified message by a vehicle in its lifetime. The trust factor of every vehicle is initially set to zero for new vehicles. This value is stored for every vehicle on the cloud before being forwarded by a fog device, which is an indicator to analyze the vehicle. Any vehicle can participate as an initiating vehicle or verifying vehicle. The trust factor is dependent on how much a vehicle correctly participates in the events. The RSU checks the validation of the message via the above-specified algorithm. If the message is proven valid, the total correct message counter (C_m_) is incremented; otherwise, the total fake message counter (F_m_) is incremented.

**Figure 11:**
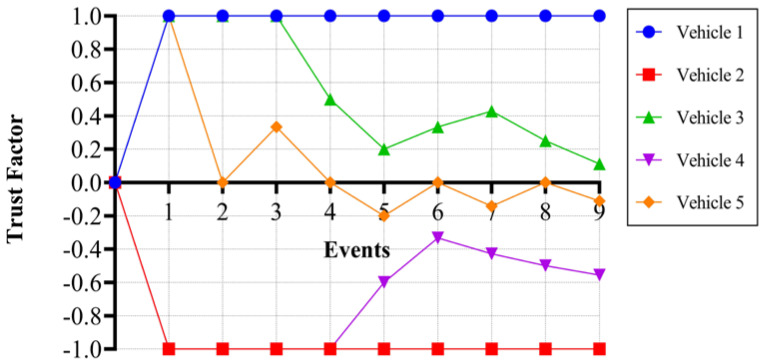
Trust factor vs. Event.

The value of T_f_ ranges from −1 to 1. A 0 value indicates that the vehicle is neutral, which means either it never participated in any communication or the number of correct and fake messages is the same. A value of one indicates that the vehicle is fully trustworthy, which means it never participated in a fake message initiation or false verification. A value of −1 indicates that the user is not at all trustworthy and has always participated in fake message initiation or false verification. A more positive value indicates that the vehicle is more trustworthy, and a more negative value indicates that the vehicle is less trustworthy. The formula to compute T_f_ is based on C_m_ and F_m_ and is shown in Equation (2). The value of T_f_ is calculated automatically when there is any change in either C_m_ or F_m_. Figure 11 depicts the deviation in the trust factor corresponding to the behavior of a vehicle.

(2) Tf=Cm−FmCm+Fm

For event message 1:

if initiated or verified correctly: T_f_ = 1 − 0/1 + 0 = 1

For event message 2:

if initiated or verified correctly: T_f_ = 2 − 0/2 + 0 = 1

For event message 3:

if initiated or verified falsely: T_f_ = 2 − 1/2 + 1 = 1/3 = 0.333

Similarly, we can calculate the trust factor for the rest of the events. To depict the behavior of a trust factor for a vehicle for either initiating a message or verifying a message, we have carried out an analysis by randomly considering messages as correct or fake. Here, we have considered five vehicles and 10 events initiated by any of the vehicles and verified it in relation to the rest of the vehicles, and the corresponding trust factor of every vehicle is calculated using the above formula. The assumption here is that each vehicle is participating in every event, which is not practical, but it is useful to show the deviation in trust factor values, as shown in Table 3.

### 5.3. Information Gain

For the message verification process, the RSU in the above algorithm sends event messages initiated by a vehicle to its nearby vehicles for verification. The RSU can decide the message’s correctness if it receives a response from at least a defined number of users, which is termed the Threshold Value (T_v_). The threshold value is dependent on the trust factor of the vehicles. If all the vehicles are completely trustworthy, then we can assume that getting a response from 25% of the total nearby vehicles would be enough for the RSU to make a correct decision. On the contrary, if the vehicles are not trustworthy, they would need more responses. The range of the threshold value is taken from a 25% to 75% response from nearby vehicles. The value of the trust factor for a vehicle ranges from −1 to 1. As per the formula given below in Equation (4), if the average trust factor, AT_f_, of nearby vehicles (calculated in Equation (3) is less than 0.25, it means the vehicles are not much more trustworthy and, hence, the RSU needs a response from at least 75% of vehicles before making a fair decision. Otherwise, it would be considering the AT_f_ for a calculation, as given below.

(3) ATf=∑i=1nTfin .

(4) Tv=ifATf≤0.25,Roundn×0.25,0Roundn×0.25ATf,0

**Figure 12:**
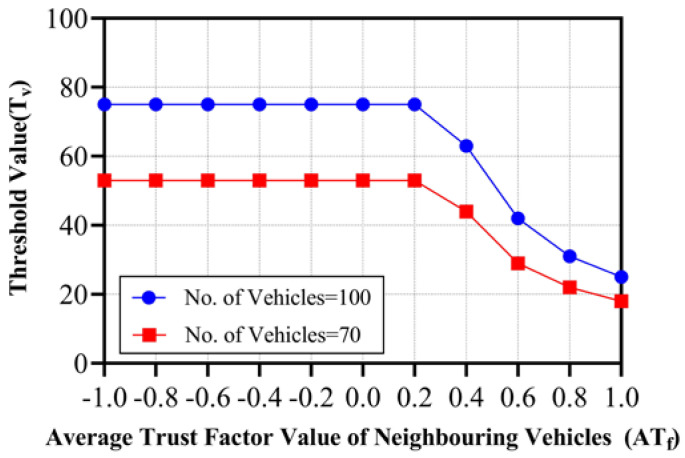
Average Trust factor (AT_f_ ) vs. Threshold value (T_v_) when Number of Vehicles are 100 and 70.

The experiment is carried out on two test scenarios, as shown in Figure 12 and Table 4, where the number of nearby vehicles is 100 in the first scenario and 70 in the second scenario. According to the formula, the T_v_ is 25% of the number of nearby vehicles if the AT_f_ of nearby vehicles is one. With the decrease in the value of AT_f_, the T_v_ will increase to 75% of the number of nearby vehicles.

## 6. Comparative Result Analysis with Implementation Details

The proposed B-SAFE architecture based on blockchain is implemented on Hyperledger Fabric, one of the popular commissioned, distributed-ledger-based platforms for blockchain implementation [47]. Hyperledger Caliper, a benchmarking tool for blockchain, is used for performance analysis. Caliper generates the throughput, latency, success and failure rate, and resource utilization in the form of HTML reports [48]. The hardware configuration of the system used for B-SAFE implementation is an Intel (R) Core (TM) i5 -1035G1 CPU@ 1.00 GB 1.19 GHz, 8 GB RAM, a 64-bit operating system, a 256 GB SSD, and 1 TB hard disk and is run on Ubuntu 16.04 LTS. The software configuration used for the implementation and performance analysis is given in Table 5. This also includes the requirements for fog nodes. Each fog node should be equipped with computing resources such as CPUs, storage, and memory to process data generated by vehicles in its proximity. These resources enable fog nodes to perform computations locally without relying heavily on centralized cloud servers. Apart from that, a compact form factor is also required to facilitate deployment in diverse environments, such as roadside cabinets, vehicles, or infrastructure poles. OpenFog, a middleware for fog computing, is also required.

**Figure 13:**
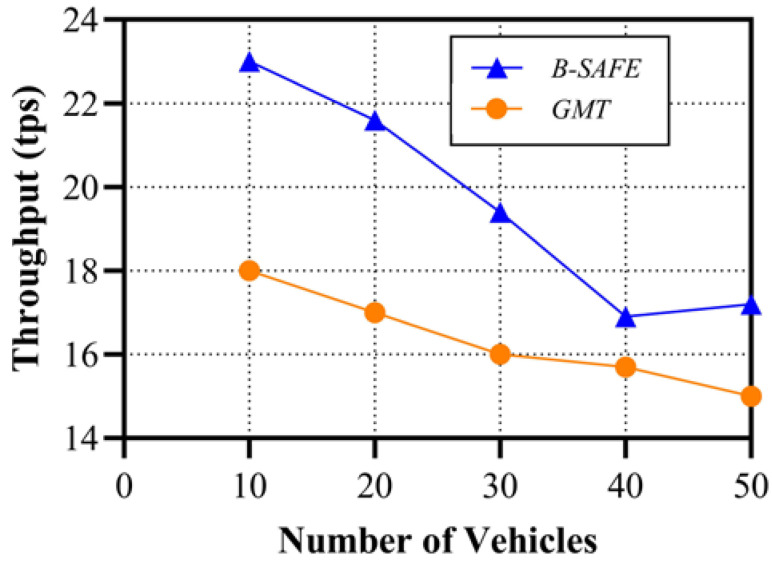
Throughput variance with different numbers of vehicles.

**Figure 14:**
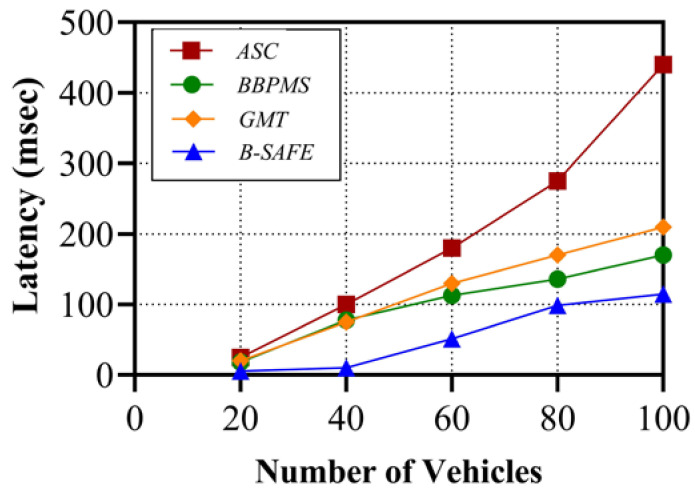
Latency variance with different numbers of vehicles.

The performance parameters analyzed for B-SAFE are throughput and related to the latency with the varying number of vehicles in the system. Throughput is evaluated in transactions per second and latency in milliseconds. The approaches considered for comparison are anonymous and require lightweight authentication based on a Smart Card (ASC) [49], a Blockchain-Based Pseudonym Management Scheme (BBPMS) for vehicular communication [50], and a General Message Transmission (GMT) protocol [51] with our proposed approach B-SAFE. The ASC method is not built on blockchain, whereas the other three approaches are. The performance comparison of B-SAFE with existing works on throughput and latency is shown in Figure 13 and Figure 14. The throughput achieved for our approach is relatively better compared to GMT. The latency of B-SAFE is the lowest compared to the other three approaches.

The high throughput and low latency are signs of the better efficiency of our proposed system. The comparison of B-SAFE and other approaches from the perspective of VANET’s security requirements is also analyzed and mentioned in Table 6.

All the compared approaches have applied a mechanism to authenticate participants in the network and keep their identity anonymous; hence, the approaches maintain the system’s privacy. Immutability, decentralization, non-repudiation, and traceability are the features of blockchain; therefore, they are satisfied by the other three approaches, except for ASC, as this approach is not based on blockchain. The data validation method is applied in ASC and B-SAFE but not in others. B-SAFE provides reward points as incentives to vehicles for their faithful involvement in the system, which is not a provision in any of the other compared systems.

## 7. Conclusions

In this work, a blockchain-enabled security architecture, B-SAFE, is presented for vehicular fog networking. The B-SAFE works faster for real-time applications when the fog computing concept is integrated. In B-SAFE, for secure and frequent communication, an RSU checks whether the event message initiated is valid or not by sending a verification message to nearby vehicles and asking them for acknowledgment. Based on the vehicle’s correctness of messages sent for initiating or verifying event messages, reward points and a trust factor are computed for each vehicle. Reward points are like credits earned, which are in the vehicle’s account and could be used later. The trust factor determines the trustworthiness of a vehicle. B-SAFE has shown to be an effective solution to timeliness, trustworthiness, and latency issues in fog-based vehicular networks. Therefore, due to this trustworthiness decision technique, the proposed B-SAFE concept improves the overall vehicular network performance. In the future, a blockchain-enabled vehicular networking framework will be realized for big-traffic-data-oriented smart traffic services. How flying drone ad hoc networks over on-road traffic can improve the performance of blockchain execution in vehicular environments will also be a focus of our future research.

## Figures and Tables

**Table 1:** Notations used in the paper.

Notation	Meaning
IV	Initiating Vehicle
EM	Event Message
L_v_	Location of Initiating Vehicle
L_e_	Location of Event
LC	Location Certificate
NBV_LIST	Nearby Vehicles List
R_c_	Response Counter
C_r_	Response saying Correct
F_r_	Response saying Fake
T	Time elapsed in the message verification process
CRV_LIST	Correct Response Vehicle’s List
FRV_LIST	Fake Response Vehicle’s List
T_v_	Threshold Value
D_t_	Defined Allotted Time
VM	Verifying Message
VN	Verifying Node
R_p_	Reward Points
C_EP_	Current Event Points
T_f_	Trust Factor for Initiating/Verifying Messages
C_m_	Total Correct message Initiated/Verified by a vehicle
F_m_	Total Fake messages Initiated/Verified by a vehicle
AT_f_	Average Trust Factor Value of nearby vehicles
IM	Initiating Message
N	Number of nearby vehicles
I	Any vehicle
T_fi_	Trust Factor for *i*-th vehicle
C	Count of messages in a block
M	Maximum messages allowed in a block

**Table 2:** Current Event Points (C_EP_) and Reward point (R_p_) for 5 Vehicles.

Event	Initiating Vehicle	Vehicle 1		Vehicle 2		Vehicle 3		Vehicle 4		Vehicle 5	
		E_p_	R_p_	E_p_	R_p_	E_p_	R_p_	E_p_	R_p_	E_p_	R_p_
1	Vehicle4	0.25	0.25	**−0.25**	−0.25	0.25	0.25	**−1**	−1	0.25	0.25
2	Vehicle2	0.25	0.5	**−1**	−1.25	0.25	0.5	**−0.25**	−1.25	**−0.25**	0
3	Vehicle3	0.25	0.75	**−0.25**	−1.5	1	1.5	**−0.25**	−1.5	0.25	0.25
4	Vehicle1	1	1.75	**−0.25**	−1.75	**−0.25**	1.25	**−0.25**	−1.75	**−0.25**	0
5	Vehicle4	0.25	2	**−0.25**	−2	**−0.25**	1	1	−0.75	**−0.25**	−0.25
6	Vehicle1	1	3	**−0.25**	−2.25	0.25	1.25	0.25	−0.5	0.25	0
7	Vehicle2	0.25	3.25	**−1**	−3.25	0.25	1.5	**−0.25**	−0.75	**−0.25**	−0.25
8	Vehicle5	0.25	3.5	**−0.25**	−3.5	**−0.25**	1.25	**−0.25**	−1	1	0.75
9	Vehicle3	0.25	3.75	**−0.25**	−3.75	**−1**	0.25	**−0.25**	−1.25	**−0.25**	0.5
10	Vehicle5	**0.25**	**4**	**−0.25**	**−4**	**0.25**	**0.5**	**−0.25**	**−1.5**	**1**	**1.5**

**Table 3:** Message Status and Trust factor (T_f_) for 5 vehicles.

Event	Vehicle 1		Vehicle 2		Vehicle 3		Vehicle 4		Vehicle 5	
	Correct /Fake	T_f_	Correct /Fake	T_f_	Correct /Fake	T_f_	Correct /Fake	T_f_	Correct /Fake	T_f_
1	Correct	1	**Fake**	−1	Correct	1	**Fake**	−1	Correct	1
2	Correct	1	**Fake**	−1	Correct	1	**Fake**	−1	**Fake**	0
3	Correct	1	**Fake**	−1	Correct	1	**Fake**	−1	Correct	0.33
4	Correct	1	**Fake**	−1	**Fake**	0.50	**Fake**	−1	**Fake**	0
5	Correct	1	**Fake**	−1	**Fake**	0.20	Correct	−0.60	**Fake**	−0.20
6	Correct	1	**Fake**	−1	Correct	0.33	Correct	−0.33	Correct	0
7	Correct	1	**Fake**	−1	Correct	0.43	**Fake**	−0.43	**Fake**	−0.14
8	Correct	1	**Fake**	−1	**Fake**	0.25	**Fake**	−0.50	Correct	0
9	Correct	1	**Fake**	−1	**Fake**	0.11	**Fake**	−0.56	**Fake**	−0.11
10	Correct	1	**Fake**	−1	Correct	0.20	**Fake**	−0.60	Correct	0

**Table 4:** Average Trust factor (AT_f_ ) and Threshold value (T_v_).

AT_f_	T_v_	
	Number of Vehicles = 100	Number of Vehicles = 70
1	25	18
0.8	31	22
0.6	42	29
0.4	63	44
0.2	75	53
0	75	53
−0.2	75	53
−0.4	75	53
−0.6	75	53
−0.8	75	53
−1	75	53

**Table 5:** Software configuration for the B-SAFE implementation.

Operating System	Ubuntu 16.04 LTS
For Client Application	HTM5.0 Jquery3.6 Golang 1.20 Web Browser–Google Chrome 121
For fog nodes	Computing resources such as CPUs, storage, and memory Compact form factor to facilitate deployment in diverse environments OpenFog (middleware for fog computing)
For Blockchain network	Hyperledger Fabric Organization1&2 ○ Peer ○ Smart contract ○ Ledger ○ MSP Orderer Channel Gossip Protocol CouchDB
For Blockchain performance evaluation	Hyperledger Caliper

**Table 6:** Software Comparison of B-SAFE with existing work.

	ASC	BBPMS	GMT	B-SAFE
Blockchain-based or not	Not based on Blockchain	Based on Blockchain	Based on Blockchain	Based on Blockchain
Implementation	VanetMobiSim, OPNET.	SUMO 0.32.0, OMNET++ 5.3, Veins 4.7.1	Ethereum, Truffle framework	Hyperledger Fabric and Caliper
Privacy	Yes	Yes	Yes	Yes
Authentication	Yes	Yes	Yes	Yes
Anonymity	Yes	Yes	Yes	Yes
Immutability	No	Yes	Yes	Yes
Decentralization	No	Yes	Yes	Yes
Non-repudiation and traceable	No	Yes	Yes	Yes
Data validation	Yes	No	No	Yes
Incentive Mechanism	No	No	No	Yes

## Data Availability

This research data will be made available on request to corresponding author for further research purpose only.
